# Grass Cutting Robot for Inclined Surfaces in Hilly and Mountainous Areas

**DOI:** 10.3390/s23010528

**Published:** 2023-01-03

**Authors:** Yuki Nishimura, Tomoyuki Yamaguchi

**Affiliations:** 1 Ph. D. Program in Empowerment Informatics, School of Integrative and Global Majors, University of Tsukuba, Tsukuba 305-8573, Japan; 2 Faculty of Engineering, Information and Systems, University of Tsukuba, Tsukuba 305-8573, Japan

**Keywords:** grass cutting, mowing, weed removal, propeller type, climbing robot

## Abstract

Grass cutting is necessary to prevent grass from diverting essential nutrients and water from crops. Usually, in hilly and mountainous areas, grass cutting is performed on steep slopes with an inclination angle of up to 60° (inclination gradient of 173%). However, such grass cutting tasks are dangerous owing to the unstable positioning of workers. For robots to perform these grass cutting tasks, slipping and falling must be prevented on inclined surfaces. In this study, a robot based on stable propeller control and four-wheel steering was developed to provide stable locomotion during grass cutting tasks. The robot was evaluated in terms of locomotion for different steering methods, straight motion on steep slopes, climbing ability, and coverage area. The results revealed that the robot was capable of navigating uneven terrains with steep slope angles. Moreover, no slipping actions that could have affected the grass cutting operations were observed. We confirmed that the proposed robot is able to cover 99.95% and 98.45% of an area on a rubber and grass slope, respectively. Finally, the robot was tested on different slopes with different angles in hilly and mountainous areas. The developed robot was able to perform the grass cutting task as expected.

## 1. Introduction

It is essential to optimally design robots aimed at replacing humans in various tasks based on the particular working environments. In particular, robots that operate in outdoor environments must be able to navigate complex, uneven terrains. Thus, in this study, we developed a robot that can work in a grassy area on a steep slope.

In recent years, agricultural robots have been increasingly developed to compensate for the declining and aging population associated with agricultural activities. Typically, the tasks and methods intended for agricultural robots may differ, as described in some studies [1,2]. This study in particular focuses on grass cutting because regular cutting of grass, referred to as weeding or mowing, is crucial in agricultural practices to prevent the grass from diverting essential nutrients and water from crops. However, human-labor-based grass cutting is often time consuming and results in low productivity. In particular, smallholder farmers in developing countries spend more than 40% of their time on grass cutting [3]. Therefore, to reduce the burden of grass cutting, grass cutting robots are being researched and commercialized. To this end, Bakker et al. proposed a grass cutting robot for organic farms [4]. Smith et al. presented a lawn mower for golf courses [5]. Daniyan et al. proposed a solar-powered lawn mower for gardens or parks [6]. Pishadory et al. developed a wireless control grass cutting robot [7]. In the context of grass cutting robots, path planning algorithms [8,9,10] and obstacle prediction [11,12,13] for robotic mowers are well studied. However, the abovementioned robots are primarily designed for flat surfaces. By contrast, Iwano et al. focused on a grass cutting robot for steep slopes in a complex environment and realized grass cutting on slopes with an inclination angle of up to 45° (inclination gradient of 100%) [14].

**Figure 1:**
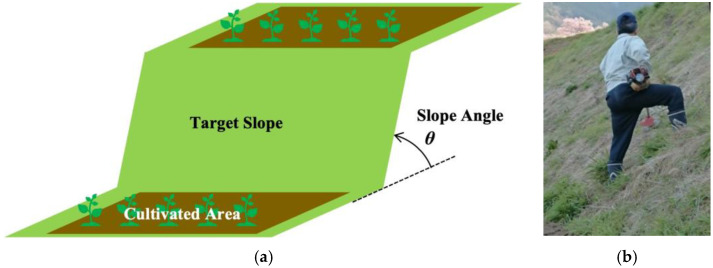
Grass cutting operations on steep slopes in mountainous areas. (**a**) Illustration of a typical gradient between cultivated areas in terraced fields in hilly and mountainous regions. (**b**) Grass cutting on a steep slope.

Notably, the robotization of grass cutting operations on steep slopes is of great importance to farmers working in mountainous areas. For example, in Japan, agricultural farmland located in mountainous areas accounts for 40% of the total farmland [15]. Moreover, grass cutting on inclined surfaces encounters additional problems because extensive labor is required, resulting in low productivity compared to that on flat land. According to a survey conducted by the Japanese Ministry of Agriculture, Forestry and Fisheries, 29.5% of the 660 grass cutting accidents reported in 2000 were caused by unstable postures of workers on steep slopes [16]. Figure 1 presents an image of a grass cutting operation on the steep slope of a cultivated field (Figure 1a); as depicted, the task is executed manually using a grass cutting machine (Figure 1b).

The survey [16] also detailed the results of field research on accidents based on interviews with farmers. Based on these interviews, we summarized 10 accidents reported during grass cutting on slopes, as shown in Table 1. From the accidents listed in Table 1, it can be observed that grass cutting on slopes is usually performed at an angle of up to 60°. Of the 10 case studies, four cases occurred due to unstable postures of workers on the slopes causing the workers to slip while holding the grass cutting machine and injure themselves. It has also been found that agricultural slopes are not regularly maintained. In three cases, accidents were reported for lands on which the grass continued to grow taller because it was not mowed regularly, and workers did not notice obstacles under their feet. As it is often difficult to restart the engine of a grass cutter on a steep slope, several accidents have been reported wherein workers halted their operations for a brief moment without stopping the engine, causing the blades to come into contact with surrounding obstacles or the workers themselves. Usually, kickback is known to occur when the rotating blade of a grass cutter comes in contact with the ground or other hard materials, and the blade bounces back toward the worker. During grass cutting operations on slopes, the blade often touches the side slopes and causes kickback. A detailed survey of the 10 cases revealed that several accidents were caused by or indirectly attributable to the particular nature of working on slopes. Owing to the frequency of accidents in such scenarios, there exists an urgent need to replace humans with robots. Therefore, in this paper, we propose a mobile robot that can perform grass cutting operations on steep slopes, and we confirm its effectiveness. The robot considered in this study is designed for grass cutting operations on slopes with an inclination of 60°. The developed robot is found to be capable of easily navigating down steep slopes while cutting the grass using a cutting device. The contributions of this study can be summarized as follows:

- We clarify the required functions for grass cutting on steep slopes. A robot with a propeller and four-wheel steering is proposed and evaluated for grass cutting on steep slopes with an inclination of up to 60° (inclination gradient of 173%).
- The observed locomotion methods are compared with the best ones for grass cutting, and the results reveal that the proposed design is suitable for grass cutting on steep slopes.
- The proposed robot is evaluated in terms of the coverage area, and the results reveal a high coverage area percentage. The developed robot is deemed capable of maintaining a stable attitude while cutting grass on slopes with an inclination of 60°.

**Table 1:** Grass cutting accidents on slopes (based on interview studies [16]).

Case No.	Age	Angle [°]	Detailed Situation of Accident
1	67	50	Unstable posture
2	76	40–50	Unstable posture
3	61	40	Unstable posture
4	57	50	Unstable posture
7	69	No Data	Kickback
9	73	15–30	Unable to detect ground owing to the presence of grass
10	35	No Data	Unable to detect ground owing to the presence of grass
11	56	32–42	Unable to detect ground owing to the presence of grass
14	68	50–60	Scattering material
16	71	No Data	Kickback

## 2. Related Studies

Slips and falls are the primary problems encountered during robot locomotion on steep slopes. In previous research, several mechanisms to maintain the stability of a mobile robot have been proposed; these include the following: (1) maintaining posture by controlling the center of gravity of a multi-legged mobile robot [17], (2) pulling a volcano observation robot with a wire [18], (3) slope movement using a snake-like robot [19], (4) traversing a slope with caterpillars or special wheels [14,20,21], and (5) maintaining posture on a slope by pushing with air [22,23,24]. In method (1), the robot must be equipped with numerous actuators to create a robot with legs, and these actuators are not only heavy but also need to be controlled in a complicated manner. In method (2), an advanced installation of wires is essential, and the robot can only move within a range that the wires can reach with no entanglement. In method (3), transporting payloads and other items is difficult compared to wheeled mobile robots. Alternatively, method (4) presents the advantage of being able to handle steep slopes by replacing the drive wheels with high friction wheels such as caterpillars or special wheels. However, the maximum angle that the robot can climb is determined based on one parameter, that is, the coefficient of friction between the slope and wheels. Therefore, we propose to use method (5) to realize grass cutting on steep slopes. This is a method that can handle steep slopes while maintaining the same control as that on a flat surface, and it can be used in combination with method (4). In method (5), the thrust force of the propeller mechanism (thrust generator) is applied to the body of the mobile robot, and this force pushes the body of the robot against the slope. Compared to the explicit use of method (4), it is possible to add the thrust force, a robot-dependent control parameter of the propellers, which allows the robot to maintain its stability on the slope, in addition to the friction coefficient between the ground and wheel, an environment-dependent parameter.

To improve the application scope of field robots, it is important that they can navigate difficult terrains and traverse steep slopes. However, in the field of mobile robots, the research on movement on steep slopes is limited. Notably, turning is one of the most difficult actions for robots to perform on steep slopes. The occurrence of slippage when turning with tracked wheels using skid steering is yet to be confirmed [14,20,21]. Skid steering is a method by which a wheeled or tracked vehicle takes a turn, and a skid steering vehicle has a greater wheel slip ratio when steering than when traveling in a straight direction [25]. When a robot turns to the right via skid steering, the left side wheels of the robot move forward, and the right-side wheels move in reverse. The phenomenon of wheel slip occurs when the grip between the wheel and surface is reduced during sharp turns or on slippery road surfaces. Although propeller thrust can be used, previous studies have not analyzed the effects of rotational motion on the design [22,23,24]. The robot may slip if the thrust force is weak. However, if the thrust force is increased, the grip of the wheels will be too strong, and the robot may not be able to turn. The other option is to use steering wheels. A robot with a steering wheel can move stably without losing grip on the ground during turning. However, a mobile robot on a slope faces another problem; that is, the robot may fall on the slope because of the change in the center of mass. Therefore, a slope robot with a Mecanum wheel is proposed [26,27]. A Mecanum wheel can perform holonomic motion on a slope; hence, there is no need to change the center of gravity with respect to the robot position. In addition, the use of a Mecanum wheel allows the robot to perform a pivot turn, resulting in minimal loss of movement. In [28], it was discovered that Mecanum wheels have a good load carrying capacity, but on an inclined or uneven surface the rim of the wheel may touch the surface instead of the roller, preventing the wheel from operating correctly. Although the robot needs to touch the ground using its wheels, Veerajagadheswar et al. presented a slope cleaning robot with Mecanum wheels in their study; this robot was able to cover more than 98% of the total area when the slope was 20° and achieved a coverage of 95.07% when the slope was 30° [26]. In a study conducted by Ransom et al., the developed robotic planetary rovers with Mecanum wheels realized locomotion on sandy terrains with a 30° slope; however, they also reported that slippage occurred when turning on a 10° slope [27]. Typically, grassy terrain can be difficult for Mecanum wheels owing to its unevenness. To achieve better performance on rough terrains, Reina et al. [29] and Qu et al. [30] proposed a four-wheel-drive/four-wheel-steer robot. The corresponding experiment indicated that the proposed robot could be used on an all terrains for agriculture. It was demonstrated that the proposed approach was effective in reducing slippage and vehicle posture errors; however, experiments on the influence of slopes are still lacking.

In previous studies, slope mobile robots focused on stabilizing attitude behavior on slopes and simple straight-line movement have been investigated; however, for performing tasks on a slope, stability when the robot changes direction is also important. Therefore, different locomotion methods need to be evaluated to determine a suitable method for slope robots. In addition, the robot must be evaluated in terms of coverage area while grass cutting on steep slopes.

## 3. Proposed Method

**Figure 2:**
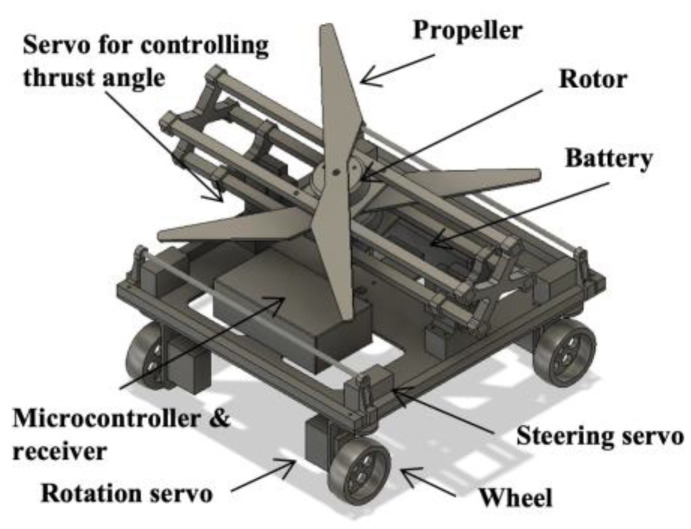
Computer-aided design of the proposed robot.

In this paper, we propose a propeller type mobile robot (see Figure 2). The propeller attached to the mobile robot is used to push the body of the robot against the slope to maintain its stability on a slope with an inclination of up to 60°. The advantage of this method is that the robot can be controlled on slopes in the same manner as on flat surfaces. Considering the stability when the robot changes direction to allow free movement on a steep slope, the effects of locomotion methods such as skid steering, two-wheel steering, Mecanum wheels, and four-wheel steering installed on the developed propeller type robot were compared.

### 3.1. Stability on a Steep Slope

**Figure 3:**
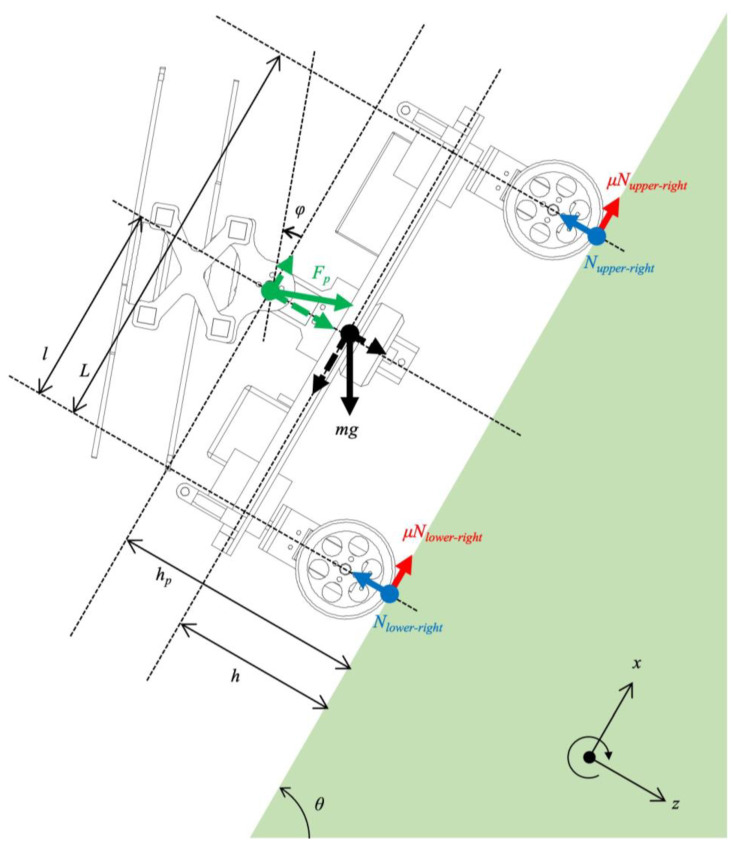
Force diagram of the proposed robot.

When mobile robots move on steep slopes, they need to overcome the lack of friction forces. In a normal situation, only gravity acts on the robot body. As shown in Figure 3, this gravity can be divided into two forces acting along the *x*-axis and *z*-axis. To avoid slipping down and maintain a stable attitude on a steep slope, the robot needs a friction force that is greater than the gravity force along the *x*-axis. Here, the friction force is the product of the normal force and friction coefficient. The normal force is along the *z*-axis and acts on the robot when it presses its body against the ground. On steep slopes, the force of gravity along the *x*-axis becomes large, and the robot requires a large frictional force. However, a large friction force is difficult to achieve on a steep slope because the normal force acting on the robot becomes small because the gravity along the *z*-axis also becomes small on a steep slope. Therefore, it is difficult to maintain a stable attitude on a steep slope without the propeller thrust force.

Figure 3 shows the force diagram of the proposed robot holding its attitude on a steep slope with the thrust of the propellers. We define the gravity acceleration as *g*, friction coefficient as *μ*, mass of robot as *m*, the slope angle as *θ*, the propeller force as $F_{p}$, and its inclination angle as ϕ.

If the proposed robot does not slip on a steep slope with angle *θ*, the friction force $F_{friction}$ acting on the robot must satisfy the following condition in Equation (1):

(1) $$F_{friction}\geq mg\sin\theta -F_{p}\sin\phi$$

As mentioned earlier, the friction force is the product of the friction coefficient and normal force, $F_{friction},$ and is written as follows:

(2) $$F_{friction}=\mu N_{upper-right}+\mu N_{upper-left}+\mu N_{lower-right}+\mu N_{lower-left}$$

$N_{upper-right}$ is the normal force acting on the wheel of the upper right side. $N_{upper-left}$,$\;N_{lower-right}$, $N_{lower-left}$ are the normal forces acting on the wheel of the upper left, lower right, and lower left wheels, respectively. In the case where the robot faces the slope head on, $N_{upper-right}$ is the same as $N_{upper-left}$, and $N_{lower-right}$ is the same as $N_{lower-left}$. These normal forces that act on the four wheels can be written as follows:

(3) $$N_{upper-right}=N_{upper-left}=\frac{1}{2}\left\{\frac{mg}{L}\left(l\cos\theta -h\sin\theta \right)+\frac{F_{p}}{L}\left(l\cos\phi +h_{p}\sin\phi \right)\right\}$$

(4) $$N_{lower-right}=N_{lower-left}=\frac{1}{2}\left\{\frac{mg}{L}\left(l\cos\theta +h\sin\theta \right)+\frac{F_{p}}{L}\left(l\cos\phi -h_{p}\sin\phi \right)\right\}$$

By substituting the *N*s in Equation (2) into Equations (3) and (4), Equation (1) is rewritten as follows:

(5) $$\mu \left(mg\cos\theta +F_{p}\cos\phi \right)\geq mg\sin\theta -F_{p}\sin\phi$$

When the wheels of the upper side leave the ground, the robot rotates backward and falls off the ground. The condition of not falling is written by using movement as follows:

(6) $$\frac{mg}{L}\left(l\cos\theta -h\sin\theta \right)+\frac{F_{p}}{L}\left(l\cos\phi +h_{p}\sin\phi \right)\geq 0$$

Considering the terms related to *mg* in Equation (6), the cosine component increases, and the sine component decreases with increasing slope angle, resulting in a decrease in the total moment when *l* > *h*. The case is limited when *l* > *h*, but this is applied for many robots. However, when *h* > *l*, the robot’s center of gravity will be high and it will fall over. Without $F_{p}$, there is a force imbalance between the *N* on the wheels of the upper slope and *N* on the wheels of the lower slope (See Equations (3) and (4)). Consequently, the magnitude of the driving force acting on the wheels on the upper and lower sides is different, and unexpected movements may occur during travel. However, it can be said that the driving force imbalance can be improved if $F_{p}$, with an inclination angle ϕ, pushes the robot body.

### 3.2. Steering Methods

**Figure 4:**
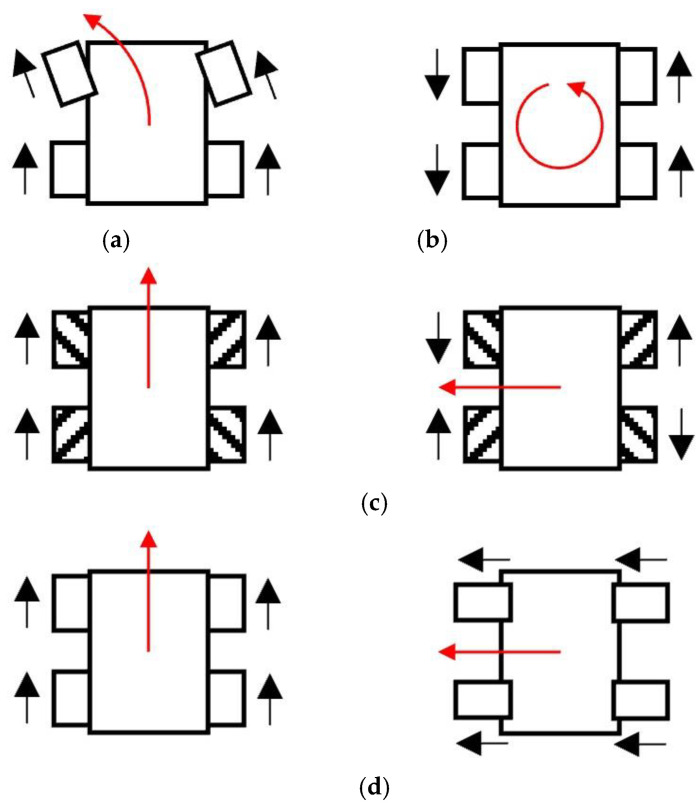
Four methods for making a left turn. The black arrow indicates the direction of the moving wheel, and the red arrow indicates the direction of the robot as a result of the moving wheels. (**a**) two-wheel steering; (**b**) skid steering; (**c**) Mecanum wheels (straight and side movement); (**d**) four-wheel steering (straight and side movement).

In this study, we tested four types of steering methods to identify the method best suited for grass cutting on steep slopes. Figure 4 shows the relationship between the direction of movement of the robot and the direction of wheel rotation, which is mainly used in this study. Figure 4a–d show the methods implemented in our robot. The four steering methods are as follows. (1) Two-wheel steering is a common method for controlling wheeled vehicles such as cars and buggies. Figure 4a shows how a robot can turn left using two-wheel steering. (2) Skid steering is used for robots with tracked wheels, similar to that presented in Iwano’s study [14]. The right-side wheels of the robot move forward and the left side wheels move in reverse when the robot makes a left turn. (3) The Mecanum wheel allows the robot to move freely by changing the direction of rotation of each wheel independently. In this manner, the Mecanum wheels can exert a driving force in both the front and side directions. (4) The robot uses four driving wheels, each of which is independently connected to steering actuators. When moving forward, the steering actuators are controlled to change the direction of the wheels in the forward direction. When moving sideways, the steering actuators are controlled to turn 90° to change the direction of the wheel sideways.

### 3.3. Strategy of Grass Cutting

To determine the design of the robot and the appropriate path of grass cutting, we ascertained the optimal type of motion for the robot to perform during grass cutting via simulation. The direction changing methods in Figure 4 can be separated into two cases. In the first case, a 90° direction change is not possible at one point, i.e., two-wheel steering. In the second case, a 90° direction change is possible, i.e., skid steering, Mecanum wheel, and four-wheel steering.

**Figure 5:**
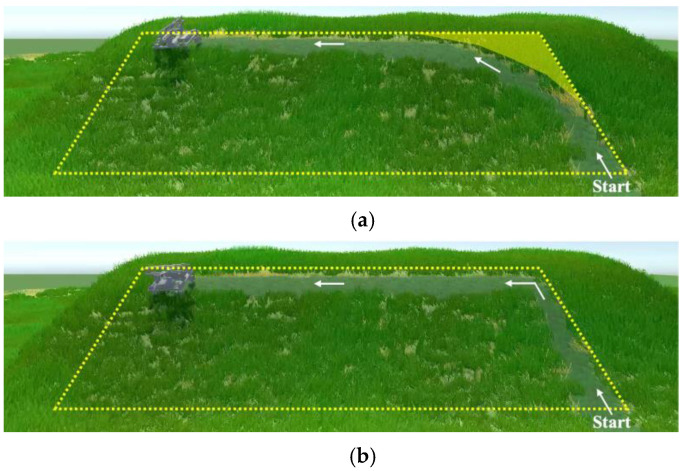
Grass cutting simulation. The inside yellow dotted line and lined arrow represent the target area and the robot moving direction, respectively. (**a**) Case 1: robot without a 90° direction change. (**b**) Case 2: robot with a 90° direction change.

For the simulation, we assumed that the robot starts working at the bottom of the slope, and it climbs up the slope as it cuts the grass. Figure 5 shows a simulation of grass cutting, where the robot cuts the grass in the target area on the slope shown inside the yellow dotted line for cases 1 and 2. In case 1, the grass remains at the corner, as shown in the yellow masked area (see Figure 5a). In the actual setting, the maximum steering angle of the wheels was 25°, and the robot length was 305 mm. Therefore, the turning radius was 0.72 m when no slippage occurred. This implies that when the robot navigated a right-angled corner, the uncovered area around the corner was 0.11 m^2^. In case 2, the robot climbs the slope and then moves sideways. Therefore, the robot can cut the grass in the corner of the target area (see Figure 5b). Although both robots are able to cover 100% of the area, the robot shown in Figure 5a will have more work to reverse through areas where the grass has already been cut to achieve a high coverage area. In comparison with the robot shown in Figure 5a, the robot shown in Figure 5b will cover 100% of the area efficiently and effectively.

In this paper, we propose using a robot that can make a 90° direction change, and we let this robot move up, down, and sideways to cut grass. From the viewpoint of mechanical energy, it is desirable to move left and right on the slope instead of moving up and down repeatedly against gravity. When grass cutting, the robot does not need to travel over the area where it has already cut the grass. Therefore, this path can reduce the overlap of the covered area and increase efficiency.

### 3.4. Developed Robot

**Figure 6:**
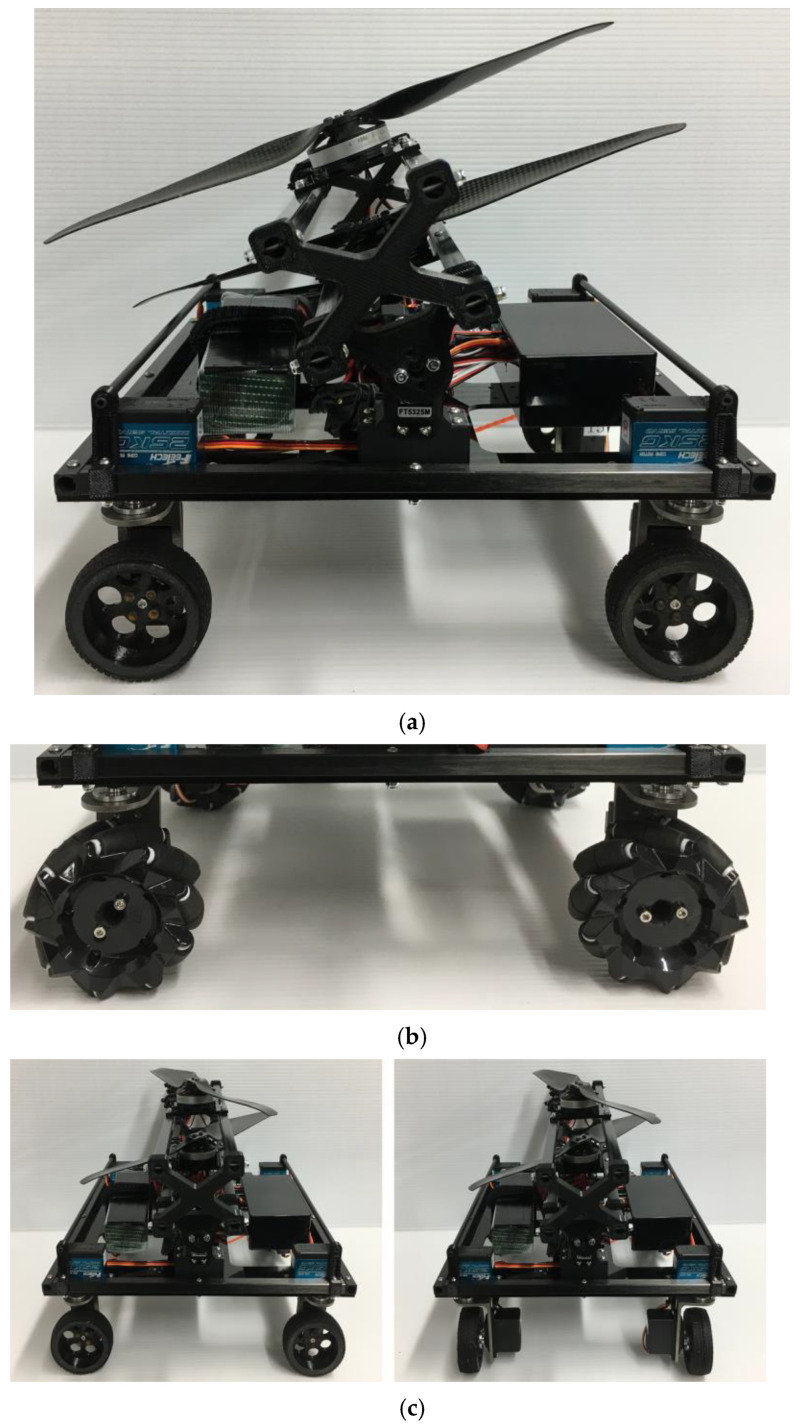

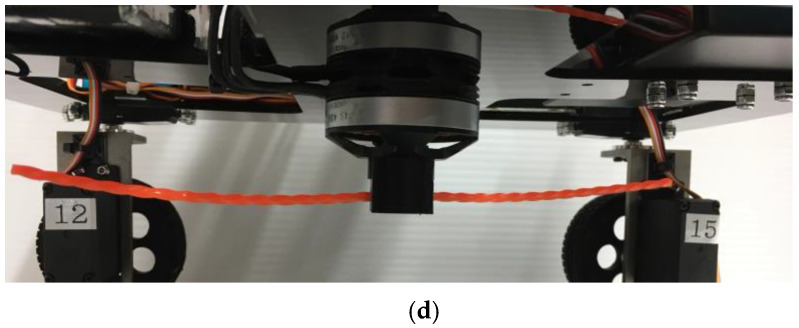
Completely developed robot. (**a**) Overview of the developed robot. (**b**) Mecanum wheel installation. (**c**) 90° rotation of the steering wheels (left: climbing mode; right: sideways mode). (**d**) Nylon cord grass cutting tool.

The developed robot is shown in Figure 6. The main frame was made of carbon pipes and the base was made of an acrylic board. Continuous rotation servomotors (FB5311M-360 from FEETECH) were used to turn the robot wheels. For steering, four servomotors (FT5325M by FEETECH) were installed to change the wheel angle (See Figure 6c). These servomotors (FT5325M from FEETECH) were also used to change the inclination of the propeller shaft. The thrust tilt angle of thrust force was driven from −20° to 20° without affecting the frame. The link between the frame and propeller, the wheels of the robot, and the base of a grass cutting module were 3D printed using polylactic acid. Three 4006/620 KV brushless rotors (TL68P02 from TAROT) were used: two for the propellers that generate thrust, and one for the grass cutting module. We used ESC (SKYWALKER 60A from HOBBYWING) to control the rotors. For the grass cutting module, we used a nylon cord to cut the grass because nylon cords are safer than rotating metal blades and can also be lighter (see Figure 6d). The size of the robot was 305 mm in length, 290 mm in width, and 242 mm in height. The weight of the robot was 2.9 kg including that of the LiPo battery (6500 mAh, 14.2 V). The robot generated a maximum thrust of 20 N with 1355 size propellers. The speed of the robot was approximately 16 cm/s. The battery had 23 min of power at medium thrust. Therefore, the robot was able to cover an area of 66 m^2^ in one operation.

This developed robot was manually controlled by an RC transmitter. The signal from the operator was transmitted wirelessly to the receiver. The microcontroller read the signal and controlled multiple servomotors simultaneously. The brushless rotors were controlled by the receiver via the ESC directly.

By substituting the real parameters, *mg* = 28.4 N, *θ* = 60°, $F_{p}$ = 20 N, ϕ = 20°, *L* = 250 mm, *l* = 125 mm, *h* = 100 mm, *h_p_* = 150 mm, *μ* = 0.92 on rubber surface and *μ* = 1.25 on grass into Equations (5) and (6), the two conditions of stable movement on steep slope were satisfied when the inclination angle was 60°. Our design was based on a stable model and was designed to work on a slope with an angle of 60°.

## 4. Experiments and Results

### 4.1. Steering Method Selection

**Figure 7:**
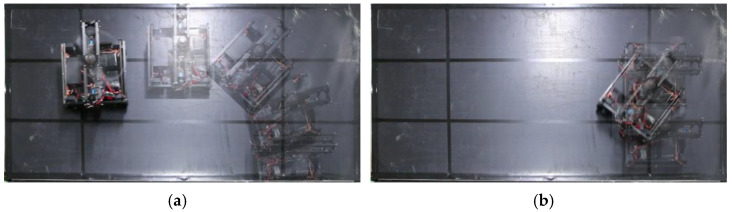

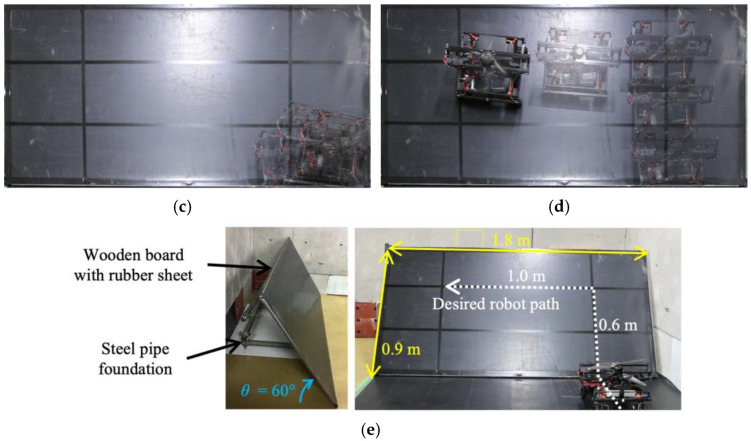
Experiment of moving on a rubber slope with four steering methods. (**a**) Two-wheel steering, (**b**) skid steering, (**c**) Mecanum wheels, (**d**) four-wheel steering, and (**e**) experimental setting.

**Figure 8:**
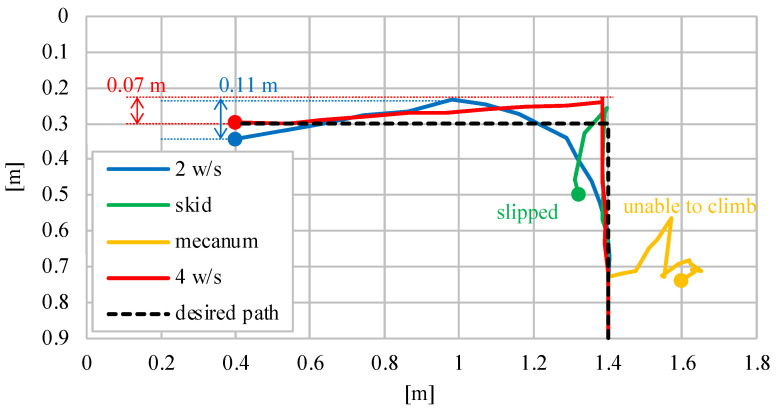
Path from the basic experiment with four types of steering methods.

First, we confirmed that the developed robot can climb, turn, and go straight on a 60° inclined rubber surface. The rubber sheet was attached to a 1.8 m × 0.9 m wooden board. After climbing 0.6 m from a flat surface, the robot made a left turn. After rotating 90°, the robot moved straight to the left until it had traveled 1.0 m. Figure 7 shows the position of the robot in 2-second intervals and Figure 8 shows the path of the robot in each case.

The two- and four-wheel steering methods were able to reach the destination point. With skid steering, the robot was able to climb the slope, but when it tried to turn, it slipped down the slope (see Figure 7b). The robot with Mecanum wheels was not able to climb slopes and was unable to reach the phase of rotation (see Figure 7c). This could be because the small free-spinning wheels of the Mecanum wheel slipped while climbing the slope. With two-wheel steering, the robot reached the target point (see Figure 7a). However, as mentioned in Section 3.3, it was unable to approach corners and edges easily. In addition, since the robot cannot make a pivot turn, it needs a large area to turn, and it may deviate from narrow slopes. The robot with four-wheel steering can move on the slope without deviating from the target path (see Figure 7d). Although there is a slight side slip, the error is not larger than that of the robot itself, so it is expected that it will not affect grass cutting. In addition, since the coefficient of friction is larger on a grassy terrain than on rubber slopes (*μ_grass_* = 1.25, *μ_rubber_* = 0.92), the effects of slippage are smaller. The slippage on grass is discussed in Section 4.3.

**Figure 9:**
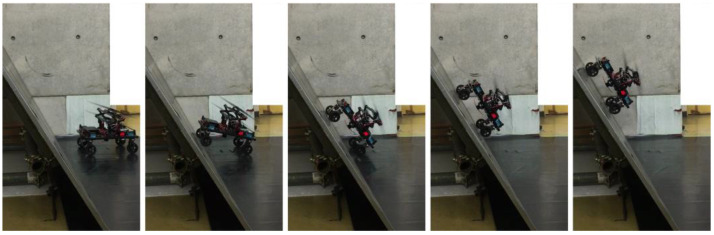
Transfer from a flat surface to a slope with an angle of 60°.

Based on the above, a grass cutting robot was realized in this study by using four-wheel steering as the suitable method for a mobile robot with a propeller mechanism. The proposed robot moving from a flat surface to an inclined surface is shown in Figure 9.

### 4.2. Coverage Area

The grass cutting work is an area-coverage task. In the basic experiment section, we used four-wheel steering to enable the holonomic movements of the robot to accomplish the area-coverage task on steep slopes. In the previous section, we validated the robot’s ability to traverse steep slopes. Therefore, we secondarily measured the coverage area while moving on steep slopes with an inclination angle of 60°.

In the study conducted by Veerajagadheswar et al. [26], the experiments were performed to validate the area-coverage capability of the developed robot at two different inclinations of 20° and 30°. They used a slope with a length of approximately 1.7 m. The experimental trials were started by placing the robot directly at the bottom of the slope. They achieved more than 98% and 95.07% coverage of the total area using their proposed method. Although this study focused on developing a robot that can move on both stairs and slopes, the robot was not tested on slopes with an angle greater than 30°.

**Figure 10:**
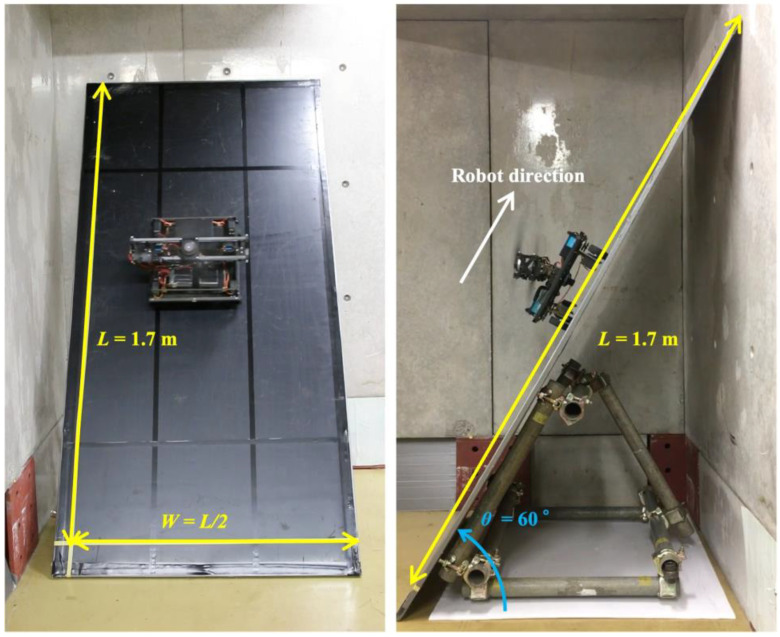
Experimental setting for the measurement of the covered area ((**left**) from front, (**right**) from side).

**Figure 11:**
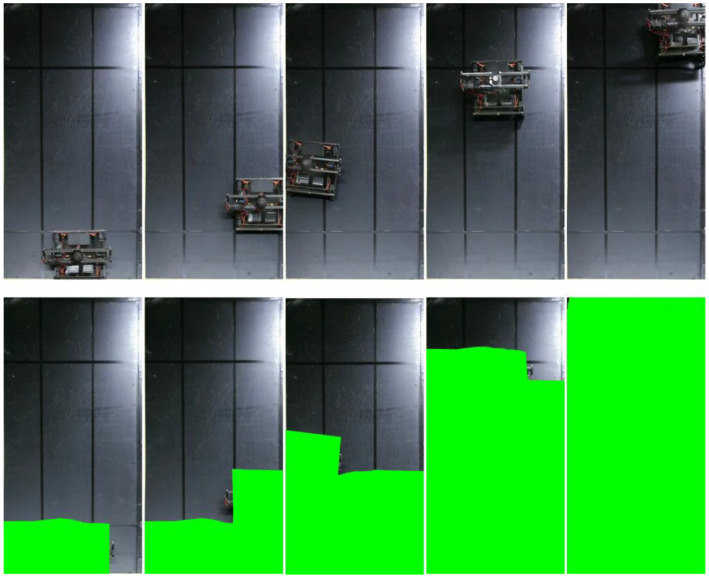
Coverage process of the robot at different points in time.

To compare the coverage area with those of previously developed slope robots, we prepared a slope with L = 1.7 m and W = L/2. The experimental setup is shown in Figure 10. We placed the robot in the initial position at the bottom left. The covered area was calculated based on the position and size of the robot and a video recording. Figure 11 shows the coverage process of the robot at different timepoints. The green area represents the total covered area. The covered area in this basic experiment was 99.95%. Although not many studies have measured the covered area, our proposed robot showed a significant improvement in the covered area on a surface with a large inclination angle. This is because our robot can realize stable locomotion in both climbing and sideways directions; hence, we conducted the experiment to measure the covered area.

### 4.3. Movement on Grassy Terrain

**Figure 12:**
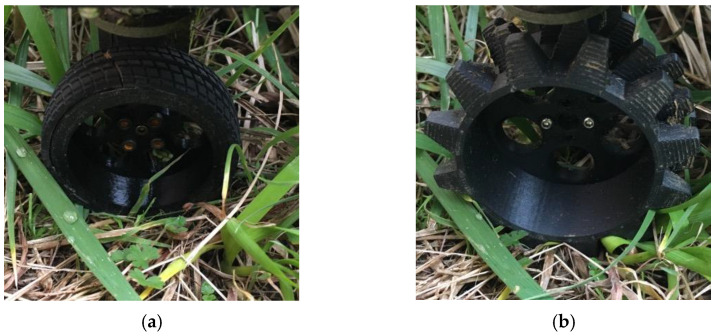
Wheels on grassy terrain. Wheel diameters are 60 mm, and the spike length is 10 mm. (**a**) Normal wheel. (**b**) Spiked wheel.

Further, we investigated how straight the robot can traverse a real, sloping grassy terrain. Iwano et al. tested their robot by moving it in a straight trajectory along a slope of 45°. As a result, their robot was able to travel a distance of 2.5 m while slipping 180 mm down [14]. We performed the same experiment on grassy terrain. The maximum slope angle on the tested terrain was 45°. First, we used the normal wheels, as shown in Figure 12a. However, the robot got stuck and could not move because the wheels were buried in the grass. To improve the movement on the grassy terrain, which is softer than the rubber surface, we changed the shape of the wheels by adding spikes, as shown in Figure 12b. The wheels had the same diameter as the base but were spiked to be able to move on grass.

**Figure 13:**
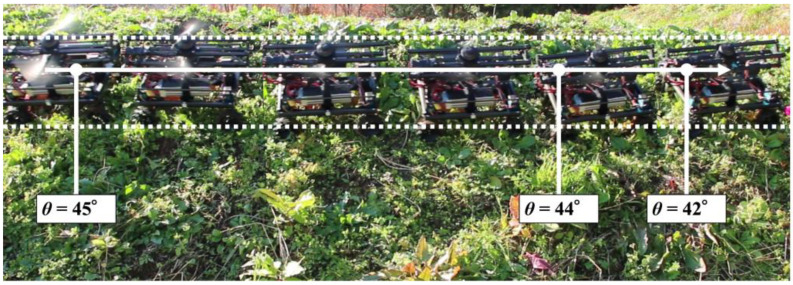
Straight movement experiment on grassy terrain in a mountainous area.

The result of the experiment can be seen in Figure 13. Our robot can travel 2.5 m without slipping even on real grassy terrain. Due to the natural terrain, the slope angle on the tested slope was not always exactly 45°. The slope was slightly curved, and there were changes in the inclination angle at the end of the movement. However, the robot was able to overcome these difficult changes of slope angle and perform stable motion. The result shows that our robot can move more stably on steep slopes.

### 4.4. Grass Cutting Experiments

**Figure 14:**
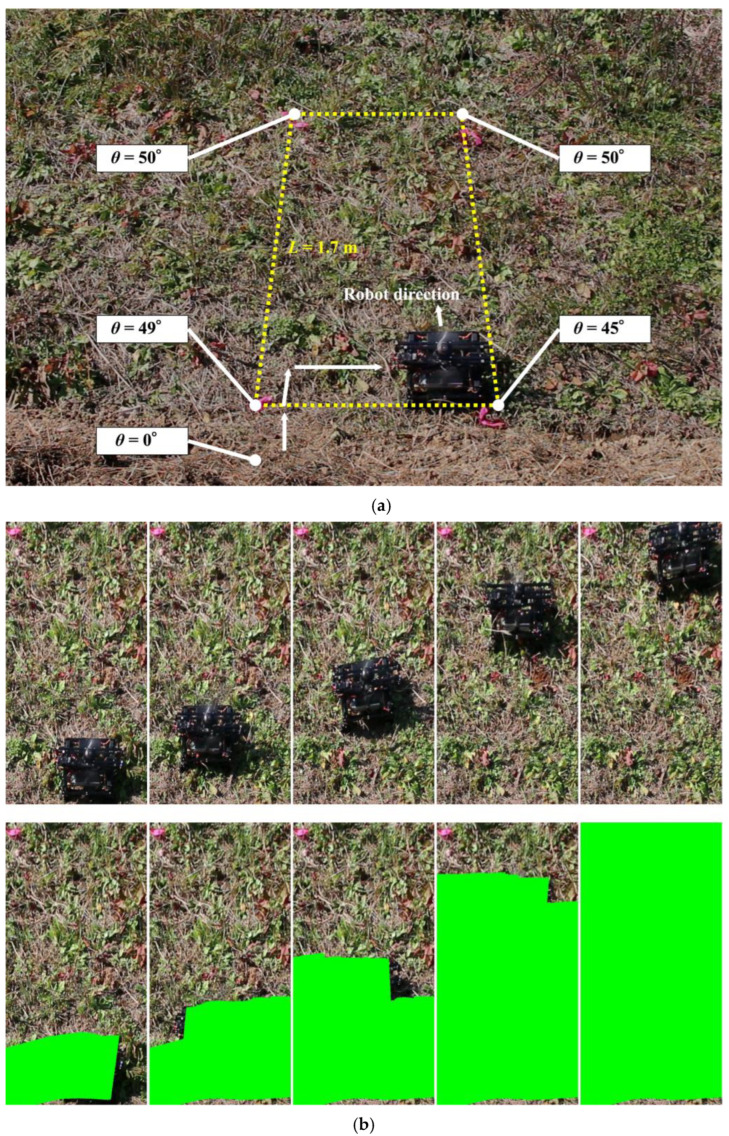
Coverage area on a grassy slope. (**a**) Target area on a slope with an inclination of 45–50°. (**b**) Coverage process of the robot at different points in time.

**Figure 15:**
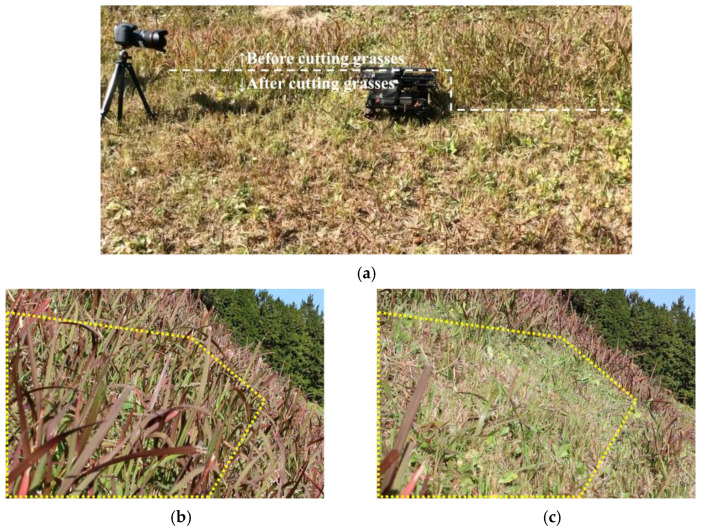
Grass cut by the robot on a slope with an inclination of 33–44°. (**a**) Experimental setting. (**b**) Before cutting the grass. (**c**) After cutting the grass.

**Figure 16:**
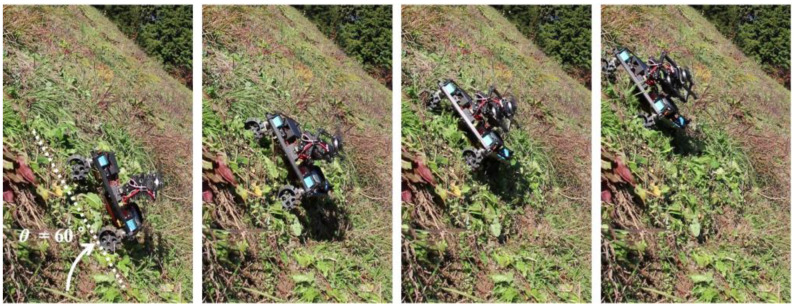
Steep slope climbing.

**Figure 17:**
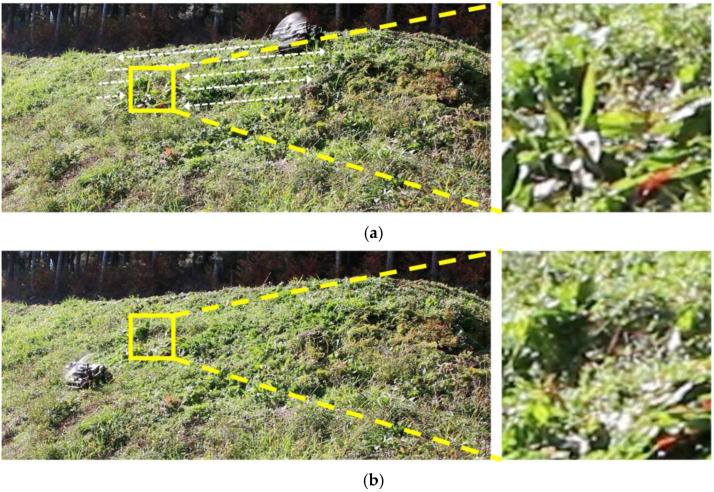
Grass cutting at different sites (slope angle 41–55°). (**a**) Before cutting the grass. (**b**) After cutting the grass.

Finally, we conducted grass cutting experiments. We experimented at different locations with different terrains and angles. The results of experiments on a real grass slope in a mountainous area are shown in Figure 14, Figure 15, Figure 16 and Figure 17.

First, we validated the covered area achieved on grassy sloping terrain as well as the rubber slope in Section 4.2. Therefore, we set the target area to L = 1.7 m and W = L/2. As markers for the target area, the anchors were embedded in the grass, and the robot was controlled from inside to cut the grass on the ground. Figure 14 shows the robot cutting grass on the slope. In the experiment, the target slope had an inclination of 45–50°. The coverage rate was 98.45% and 99.95% on the grassy and rubber terrain, respectively. There was an uncovered area around the base of the square. Although the robot was controlled to move on the baseline of the target square, position errors occurred because it moved on the edge where the slope angle changed from a flat surface with an angle of 0° to a slope with an angle of 49°.

**Figure 18:**
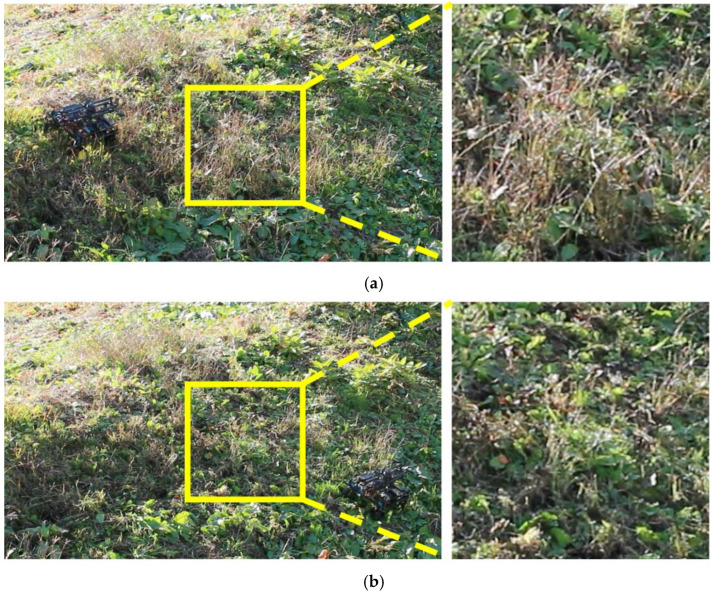
Grass cutting at different sites (slope angle 18–40°). (**a**) Before cutting the grass. (**b**) After cutting the grass.

Next, we verified that the grass was properly cut by the developed robot. Figure 15 shows a side view of the robot cutting grass on a slope with an inclination of 33–44°. From Figure 15, it can be seen that the grass with a length of approximately 30 cm has been cut to a length of 5 cm in the area covered by the robot. Based on the above two results of the covered area percentage and grass cutting performance, it was confirmed that the proposed mobile robot can perform grass cutting operations on steep slopes. Figure 16 shows a slope with an inclination of 60°. The robot was able to maintain attitude, climb the slope, and move along the slope without falling or slipping. The slope angle of 60° was the maximum slope angle found in this experiment. As summarized in Table 1, we confirmed that grass cutting operations at 60° can be performed by the robot developed in this study. Figure 17 and Figure 18 show grass cutting on a typical slope in a mountainous area. There is cultivated land on the upper and lower sides of the slope. As depicted in Figure 17, the robot started at the upper side of the slope and continued working while moving left and right to the lower side. We can observe that the large grass leaf in the lined square area was also cut. On the slope in Figure 18, the robot moved from the upper left side of the slope to the lower right side of the slope. This experimental environment had an angular range of 18° to 40°. We have confirmed that our proposed robot can overcome the angular change on steep slopes on uneven terrain.

## 5. Discussion

In the steering methods experiment, two-wheel steering, skid steering, Mecanum wheels, and four-wheel steering were proposed and compared as locomotion methods for a steep slope grass cutting robot. A four-wheel steering robot was able to move freely on steep slopes by changing the direction of wheel rotation with connected servo motors. The Mecanum wheels were able to operate on gentle slopes when the surface is flat as studied in a previous article, but cannot be used on steep slopes because the wheels are too weak to handle uneven surfaces. Therefore, we considered four-wheel steering to be the best method when the robot is to perform a task such as cutting grass on a sloped surface.

It was found that a 90° turn was important for the robot performing the area-coverage task, as it reduced the non-covered area around the edges. The coverage area was calculated when moving on a steep slope. The result shows a coverage of 99.95%. As shown in Figure 11, the area not covered was the top corner of the wooden panel, which was difficult to reach because the robot should not fall off the edge of the panel for safety reasons. However, the robot can be controlled to not fall off the surface during the task. The robot covered 98.45% of the grass terrain area while grass cutting, as shown in Figure 14. The percentage of area covered is similar to the study by Veerajagadheswar et al. [26] and differs only slightly on a flat rubber surface and a grassy steep slope. The combination of propeller-based attitude stabilization and four-wheel steering allows the robot to cover the same area as on a flat surface, including grass cutting on a steep slope. Thus, this robot does not require any special control such as trajectory correction when the thrust force pushes the robot against the ground as shown in Figure 13.

In the final experiments, the grass cutting operations were properly performed by the developed robot. The rotating nylon cord shows its ability to cut the grass. Compared with using a metal blade, the nylon cord is safer because it can only cut soft material such as grass but cannot seriously injure people or damage property. In addition, the nylon cord is very light compared to metal blades. This has the advantage of reducing the mass of the robot and allowing it to navigate steeper slopes. In addition, the inertia of the rotating nylon cord is much lower than the inertia of the rotating metal blade.

We confirmed the possibility of using our robot in mountainous agricultural areas. We tested the grass cutting robot on different kinds of slopes with slope angles ranging from 18° to 60° on a real terraced field. The wheel shape we first used did not work well because the wheel slipped on the grass. After updating the wheel shape with spikes, the robot moved well on the grassy terrain. The steering methods were investigated in this study, but the shape of the wheel also needs to be analyzed to achieve a more stable movement on the slope. The results confirm that the robot proposed and developed in this study is capable of cutting grass on steep slopes in hilly and mountainous areas, with a coverage rate of 98.45%. We also confirmed that the proposed robot can move on 60° slopes in the actual environment. Since the developed robot can navigate uneven terrain with steep angles, the proposed robot with a propeller and four-wheel steering can be used for various tasks such as cleaning and maintenance of buildings on slopes.

## 6. Conclusions

Grass cutting in mountainous areas is a complex task performed on uneven terrain with steep slopes. Farmers cut grass on steep slopes with an angle of up to 60°, and serious accidents have been reported because of falls while holding a rotating blade. In this study, a novel grass cutting robot was presented. We developed a mobile robot equipped with four-wheel steering and a propeller to provide stable locomotion on steep slopes while cutting grass (a video demonstrating grass cutting experiments was provided in the Supplementary Materials). The developed robot is capable of locomotion in a real environment and can cut grass on steep slopes. The results were compared with existing studies in terms of slippage distance during straight motion and coverage area percentages. We also conducted grass cutting experiments on different slopes, and found that the grass was cut as expected. Since the proposed robot with a propeller and four-wheel steering is capable of traversing uneven terrain with steep angles, this method can be used for various types of area-coverage tasks such as cleaning and maintenance of sloped structures.

In future work, automation of grass cutting with the proposed robot will be considered. The robot must avoid stones and wood sticks as obstacles when cutting the grass. Therefore, obstacle detection using a camera as a sensor will be investigated. Additionally, in hilly and mountainous areas, the robot needs to move from one terrain to another in terraced fields because not all target slopes connect to one another. In this case, the robot must move on low inclination slopes as well as flat surfaces. Yang et al. have been presented a robotic mower with Inertial Measurement Unit (IMU) [31]. Therefore, in order to control thrust magnitude and direction, research on the use of IMU to measure the slope angle is required. Additionally, the robot must move through an entire terraced field. Therefore, the use of self-localization using Global Navigation Satellite Systems (GNSS) such as presented in Martelloni et al.’s study [32] must also be implemented. Finally, path decision, which will allow the system to perform autonomous grass cutting, will be investigated.

Although our robot showed significant improvement in slope angle in this paper, autonomous navigation, grass cutting ability, maximum slope angle, working capacity should be compared with commercial products toward the practical application of the proposed robot.

## Data Availability

Not applicable.

## Supplementary Materials

The following are available online at https://www.mdpi.com/article/10.3390/s23010528/s1, Video S1: Grass cutting experiments in hilly and mountainous area.
